# (Pre-) atherosclerotic vessel changes in patients with chronic obstructive pulmonary disease and eosinophilia

**DOI:** 10.1186/s12890-025-03720-y

**Published:** 2025-06-02

**Authors:** Leonie Biener, Janne Carolin Drews, Carmen Pizarro, Max Jonathan Stumpf, Nadjib Schahab, Christian Schaefer, Dirk Skowasch

**Affiliations:** Department of Internal Medicine – II, Cardiology, Pneumology and Angiology, University of Bonn, Venusberg Campus 1, 53127 Bonn, Germany

**Keywords:** COPD, Blood eosinophil count, Strain analysis, Atherosclerosis

## Abstract

**Background:**

Chronic obstructive pulmonary disease (COPD) is accompanied by systemic inflammation and an increased risk of cardiovascular diseases, including atherosclerosis and abdominal aortic aneurysms. Eosinophilic inflammation is common in COPD, but little is known about the role of eosinophilia in atherogenesis.

**Objective:**

The study aims to investigate a possible link between the blood eosinophil count (BEC) in stable COPD patients and arterial vessel changes of the infrarenal abdominal aorta (AA) and common carotid arteries (CCAs).

**Methods:**

One hundred seven patients were acquired. Ultrasonography imaging was employed to assess atherosclerotic plaques and AA diameter, vascular speckle tracking was used to evaluate vessel movement by vascular strains of the AA and CCAs. Patients were divided into two groups, comparing a low (< 300/µl) and high (≥ 300/µl) BEC. The circumferential (rad.) strains and aortic diameter were defined as primary outcome measures.

**Results:**

The strains values of the left and right CCA did not differ between the groups (left CCA: 3.0 ± 1.6% vs. 3.6 ± 1.5%, *p* = .053, right CCA: 3.5 ± 1.8% vs. 4.1 ± 1.8%, *p* = .127), neither did the aortic diameter (1.88 ± 0.8 vs. 1.79 ± 0.8 cm, *p* = .674) or atherosclerotic plaque burden. There were lower strain values of the abdominal aorta (3.6 ± 1.5 vs. 2.8 ± 1.4, *p* = .014), reduced radial displacement (0.16 ± 0.1 vs. 0.11 ± 0.1 mm, *p* = .011) and an association of BEC and strain values in linear regression analysis (b = -0.001 [95% CI: -0.003–0.001], *p* = .044), indicating an impaired vascular movement. However, it could not detect an association between BEC and strains of the CCAs (*p* = .664 resp. .576) or the aortic diameter (*p* = .672).

**Conclusion:**

The study shows no persuasive association between BEC in COPD and vascular strain values or aortic diameter. However, BEC was associated with reduced movement of the AA.

## Introduction

Chronic obstructive pulmonary disease (COPD) is a heterogeneous lung condition primarily caused by cigarette smoking, and is recognized by the World Health Organization (WHO) as the third leading cause of death worldwide [1]. Importantly, approximately 30% of deaths among COPD patients are attributed to cardiovascular causes [2, 3]. Cardiovascular complications are frequently observed in COPD patients beyond the shared risk factors such as smoking [4]. Several contributing factors have been identified, with systemic inflammation being a key component [3, 5]. The inflammation, which is aggravated during acute exacerbations, facilitates the development of cardiovascular complications, including atherosclerosis and its sequelae.

Recent studies have suggested an association between the blood eosinophil count (BEC) and the risk of atherosclerosis and abdominal aortic aneurysms [6, 7]. We already found an association of BEC and increased abdominal aortic diameter and reduced vascular strain values in asthma [8, 9].Approximately 20–40% of COPD patients exhibit eosinophilic inflammation [10]. However, the potential role of eosinophilic granulocytes as a pathomechanistic link between COPD and vascular pathologies remain unclear. Therefore, our research aims to determine whether elevated eosinophil counts in COPD patients are associated with a reduced strain values, aortic diameter, or the presence of vascular plaques, which we summarized as (pre-)atherosclerotic vascular changes.

## Material and methods

### Study design

**Fig. 1:**
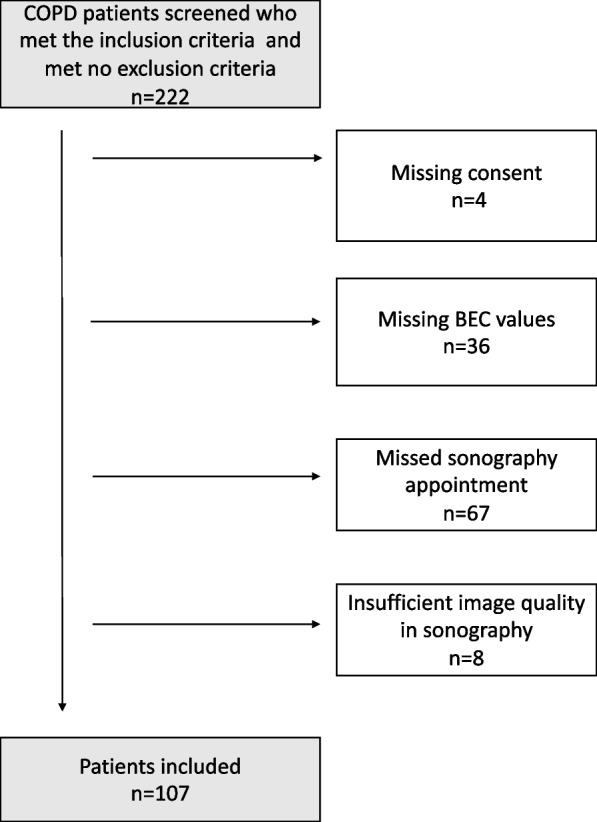
Flow-chart patient inclusion

This explorative prospective cohort study was conducted between July 2023 and April 2024 at the Outpatient Department of Pneumology and Angiology of the University Hospital of Bonn. To include as many patients as possible, we screened all patients within the inclusion period, of which 222 patients fulfilled the inclusion criteria of a pre-diagnosed condition of COPD who were over the age of 18, exclusion criteria included pre-diagnosed eosinophilic asthma and comorbidities associated with elevated BEC, such as hypereosinophilic syndrome and eosinophilic granulomatosis with polyangiitis (EGPA). Patients who were unable to give consent were also excluded from the study. Of all patients screened, 4 were excluded for missing consent, 36 for missing BEC values, 67 missed the sonography appointment and 8 had an insufficient image quality of sonography (depicted in figure 1). 107 patients were finally included to the final analysis, and divided into two subgroups stratified by their BEC (high BEC group with ≥300/µl and low BEC group with <300/µl). We additionally performed a subgroup analysis of patients with COPD group E, who are at a specific risk for cardiovascular events. All patients were in a stable condition without a current exacerbation at the time of the examination.

### Ethical statement

This study protocol was approved by the local ethics committee of the Medical Faculty of the University of Bonn, Rheinische-Friedrich-Wilhelms-Universität Bonn, under file No. 117/22. The study was conducted in accordance with the principles outlined in the Declaration of Helsinki. All participants provided written informed consent.

### Data collection

Information regarding the patients’ medical conditions and history, including current medications and BEC levels, was obtained from their medical records. Pulmonary function tests (PFTs) were conducted during their outpatient consultations as part of the standard clinical routine. The only additional examination performed for this study was color-coded duplex sonography.

### Vascular imaging

Following a regular check-up at the pulmonary department, the same day a vascular examination was conducted by experienced examiners who were blinded to the patients'blood eosinophil count results.

This examination was performed with a Philips EPIQ2 ultrasonic device, the infrarenal aorta was scanned with an abdominal probe C5-1, cerebral arteries with a linear probe L12-3. A complete cerebrovascular screening (including the common carotid arteries, proximal internal carotid artery, external carotid artery, vertebral arteries) and examination of the abdominal aorta were performed. B-mode, color-coded duplex sonography and pulse waved doppler sonography were used for all vessels. The vessels were examinate in long- and short-axis view. The loops for strain analysis were performed in short-axis view one centimeter below the carotid bulb [11].

The diameter of the abdominal aorta (AA) was measured manually from the infrarenal aorta, using the leading-to-leading edge method [12, 13]. This procedure was performed because of its low- cost and high accuracy as a tool for screening and diagnosing aortic aneurysms, with high sensitivity (94–100%) and specificity (98–100%) [14, 15]. As per the ESC guidelines on the diagnosis and treatment of aortic diseases, an abdominal aorta diameter ≥ 3 cm was classified as pathological [16].

Atherosclerosis was defined as a combined endpoint of any atherosclerotic plaque, which in turn was defined as follows: plaque of the cervical arteries was present by meeting two criteria out of three (intima-media thickness > 1.5 mm; protrusion into the lumen; abnormal wall texture), while aortic plaque was defined according to lower extremity arteries (abnormal protrusion into the lumen and/or abnormal wall texture), according to Stumpf et al. [17].

**Fig. 2:**
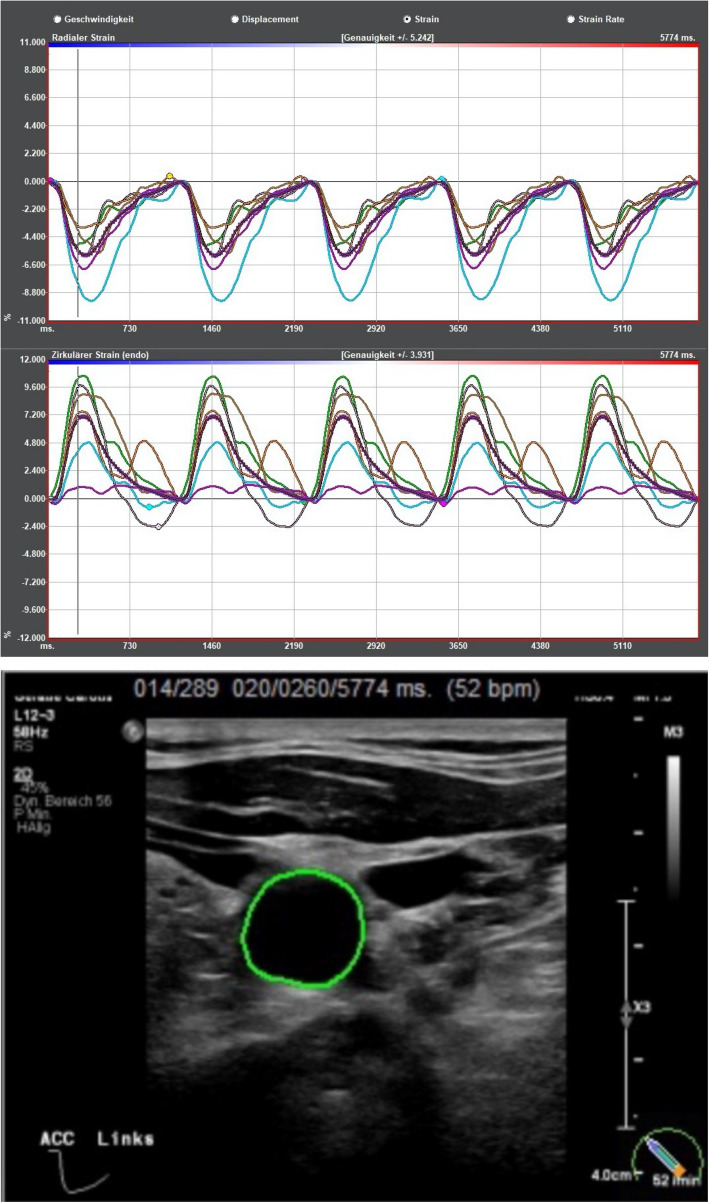
**a** Example of a strain analysis (upper lines: “Radialer Strain” = circumferential (rad.) strain, lower lines: “Zirkulärer Strain” = circumferential strain), carried out with ImageArena® Version 4.6 software from TomTec Systems GmbH in Munich, Germany. **b** The green area around the vessel on the left represents the tracking of the radial expansion of the left common carotid artery

The examination was complemented with a videotape of the cross-sectional area (CCA and AA) during five cardiac cycles in B-mode, triggered by electrocardiography (ECG). During this short time patients were asked to hold their breath while the internal jugular vein was compressed. ImageArena® Version 4.6 software from TomTec Systems GmbH in Munich, Germany, was used to gather and analyze data for the strain analysis [11, 18, 19]. The circumference of the vessel wall was marked manually along the intima-media complex, the tracking of the vessel wall motion during the cardiac cycle and calculation of the strain values is done automatically by ImageArena® (see Fig. 2 a + b). Multivarious data including circumferential strain (change of the vessel wall circumference during one heart cycle in %), circumferential (rad.) strain (change of the vessel wall during one cardiac cycle in % based on radius, in other publications referred to as radial strain [9, 11, 20–22], radial strain rate and circumferential strain rate (dynamic parameters of the vessel wall motion over time (1/s)), radial displacement (overall movement of the vessel wall as movement of a speckle averaged over all segments in millimeter (mm);) and radial velocity in centimeters per second (cm/s) were collected.

Vascluar speckle tracking is used to measure vessel wall motion and vascular stiffness [23, 24]. To outline the technique, Fig. 2 a and b) depict an example of the output of a speckle tracking based strain analysis. Lower values of the radial strain imply lower vascular movement, carotid circumferential strain and strain rate are independent predictors of major cardio- and cerebrovascular events in patients without established cardiovascular disease, and may therefore indicate the presence of (early) atherosclerosis [21, 23–25].

The circumferential (rad.) strains of all vessels and the diameter of the abdominal aorta were defined as primary outcome variables.

### Blood parameters/laboratory testing

All blood values, including the count of eosinophil granulocytes, leukocytes, alpha-1-antitrypsin levels, and C-reactive protein (CRP), were collected from EDTA-blood and serum samples. These samples were obtained as part of the standard clinical routine. Patients consented to the use of these blood values from their medical history files. The most recent blood eosinophil count (BEC) was recorded when the patient was not experiencing an acute exacerbation and had not undergone systemic corticosteroid treatment.

### Pulmonary function test and blood gas analysis

All pulmonary function test (PFT) values were obtained from measurements during routine clinical practice. PFT was performed with Vyaire Medical Vyntus™ BODY Plethysmpgraph. Blood gas analysis (BGA) was conducted under room air conditions, or with the use of oxygen supplementation in patients undergoing long-term oxygen therapy (LTOT), as part of the routine clinical practice (Siemens Healthcare, RapidLab® 1265 Blood Gas Analyzer).

### COPD

The spirometric diagnosis of COPD was established following the American Thoracic Society (ATS)/European Respiratory Society (ERS) recommendations and the German National Clinical Practice Guideline (NVL), using the thresholds recommended by the Global Lung Initiative (GLI) [26–28]. Accordingly, the COPD grades were classified based on the impairment of forced expiratory volume in one second (FEV1), and the A, B, and E groups were categorized according to symptoms and exacerbations.

To evaluate health-related quality of life (QoL), we used the COPD assessment test (CAT) as a self-administered, validated questionnaire with 8 items. The score can reach from 0–40 points, with more points indicating a worse QoL, a value of less than 10 is considered a low symptom burden [29, 30].

### Statistical analysis

The data were analyzed using IBM® SPSS® Statistics, Version 29.0. Descriptive statistics are presented as n (%) for categorical variables, and as means ± standard deviation (SD) for continuous variables. Normal distribution was assessed graphically. Non-normally distributed continuous variables are presented as median [25%-percentile;75%-percentile].

Categorical data were compared using the Chi-Square test or Fisher's exact test, as appropriate.

We compared two groups with an either high (≥ 300/µl) or low (< 300/µl) BEC. For comparing continuous variables between groups, an unpaired Student’s t-test was used. In cases where the Levene test indicated heterogeneity of variances, the Welch test was employed. For non-normally distributed data, non-parametric Mann–Whitney U test was applied.

Given the small sample sizes in the subgroups, medians with interquartile ranges (IQR) were reported, and non-parametric tests were utilized for subgroup analyses.

To further assess the relation of BEC and the primary outcome variables, linear regression analysis was used, adjusted for the usual cardiovascular risk factors (pack-years, diabetes mellitus type 2, obesity, dyslipidemia, family history).

A p-value of less than 0.05 was considered statistically significant.

The specific statistical tests used are indicated in the result tables. Missing values due to incomplete data are also reported in the result tables. Due to the exploratory nature of the study aimed at generating hypotheses and the already non-significant *p*-values, we abstained from correction for multiple testing.

## Results

### Patients ‘ characteristics

Baseline characteristics are presented in Table 1. The study included 83 patients included with a BEC of < 300/µl and 24 patients with a BEC of ≥ 300/µl. The majority of patients were male in both groups (67.5% and 75.0%, respectively *p* = 0.482). Patients in the BEC ≥ 300/µl group had a higher number of pack-years, although both groups demonstrated a pronounced smoking history (median of 40 [30;50] vs. 50 [40;60], *p* = 0.028). There were no differences in sex category, age, or cardiovascular risk factors between the groups.

**Table 1:** Baseline characteristics

**Variables**	***n* overall**	**BEC < 300 n/µl *n* = 83**	**BEC ≥ 300 n/µl *n* = 24**	***p*-value**
Sex, female	107	27 (32.5%)	6 (25.0%)	.482 ^a^
Age (years)	107	66.7 ± 10.6	67.3 ± 7.8	.780 ^c^
BMI (kg/m^2^)	107	25.8 ± 5.1	26.6 ± 5.1	.496^c^
Dyslipidemia	107	24 (28.9%)	7 (29.2%)	.981 ^a^
Diabetes mellitus type II	107	10 (12.0%)	3 (12.5%)	1.000 ^b^
Arterial hypertension	107	41 (49.4%)	13 (54.2%)	.681 ^a^
Systolic blood pressure (mmHg)	89	126.8 ± 18.2	133.2 ± 22.4	.177 ^c^
Diastolic blood pressure (mmHg)	89	79.0 ± 4.3	73.7 ± 12.6	.122 ^c^
Family history of CVD	107	10 (12.0%)	1 (4.2%)	.450 ^b^
Smoking status				1.000 ^b^
Active smoker	107	20 (24.1%)	5 (20.8%)	
Ex-smoker		60 (72.3%)	18 (75.0%)	
Never-smoker		3 (3.6%)	1 (4.2%)	
Pack years	104	40.0 [30.0;50.0]	50.0 [40.0;60.0]	.028* ^e^
**COPD**				
COPD grade	107			.155 ^b^
GOLD 1		7 (8.4%)	0 (0.0%)	
GOLD 2		44 (53.0%)	9 (37.5%)	
GOLD 3		22 (26.5%)	10 (41.7%)	
GOLD 4		10 (12.0%)	5 (20.8%)	
GOLD group	107			.293 ^b^
A		18 (21.7%)	4 (16.7%)	
B		39 (47.0%)	8 (33.3%)	
E		26 (31.3%)	12 (50.0%)	
A + B vs. E		57 (68.7%) vs. 26 (31.3%)	12 (50%) vs. 12 (50%)	.092 ^a^
Exacerbations in last 12 months	107	0 [0;1]	0 [0;1.75]	.603 ^e^
Time since last exacerbation (months)	107	1 [0;8]	2.5 [0;11.5]	.278 ^e^
CAT score	91	20.6 ± 8.6	23.4 ± 7.9	.204 ^c^
**Medication**				
LAMA	107	78 (94.0%)	23 (95.8%)	1.000 ^b^
LABA	107	78 (94.0%)	23 (95.8%)	1.000 ^b^
ICS	107	36 (43.4%)	16 (66.7%)	.004* ^a^
Roflumilast	107	5 (6.0%)	3 (12.5%)	.375 ^b^
Azithromycin prophylaxis	107	3 (3.6%)	0 (0.0%)	1.000 ^b^
LTOT	107	25 (30.1%)	8 (33.3%)	.764 ^a^
**Comorbidities**				
Coronary heart disease	106	27 (32.9%)	9 (37.5%)	.677^a^
OSAS	107	8 (9.6%)	2 (8.3%)	1.000 ^b^
Pulmonary hypertension	107	13 (15.7%)	3 (12.5%	1.000 ^b^
AAT deficiency	81	4 (6.3%)	0 (0.0%)	.574 ^a^

Categorial variables are presented as n (%). Metric variables are presented as mean ± standard deviation or median [25%-percentile;75%-percentile] when not normally distributed

*Abbreviations*: *AAT* Alpha-1 antitrypsin deficiency, *BEC* blood eosinophil count, *BMI* body-mass index, *CAT* COPD assessment test, *COPD* chronic obstructive pulmonary disease, *CVD* cardiovascular disease, *ICS* inhaled corticosteroid, *GOLD* Global Initiative for Chronic Obstructive Lung Disease, *LAMA* long-acting muscarinic antagonist, LABA: long-acting beta-2-agonist, LTOT: long-term oxygen therapy, µl: microliter, OSAS: obstructive sleep apnea syndrome

^*^p <.05

^a^chi-square-test

^b^Fisher’s exact test

^c^unpaired t-test

^d^Welch test

^e^Mann-Whitney-U-test

COPD severity was comparable in both groups concerning GOLD grade, with most patients classified as GOLD 2 or 3 (53.0% and 26.5% vs. 37.5% and 41.7%, *p* = 0.155), and a substantial proportion of Group E patients in each group (31.3% and 50.0%, *p* = 0.293). The symptom burden was similar between groups (CAT score 20.6 ± 8.6 vs. 23.4 ± 7.9 *p* = 0.204), as the overall annual exacerbation rate (median of 0 [0;1] vs. 0 [0;1.75], *p* = 0.603).

Regarding medication, most patients were prescribed a long-acting muscarinic antagonist (LAMA) and a long-acting beta-2-agonist (LABA), a higher proportion of patients in the high BEC group were also receiving an inhaled corticosteroid (ICS, 43.4 vs. 66.7%, *p* = 0.004).

The results of pulmonary function tests and laboratory testing are displayed in Table 2. Patients with a BEC ≥ 300/µl exhibited slightly more impaired pulmonary function compared to those with a BEC < 300/µl, as indicated by forced expiratory volume in 1 s (FEV1% predicted, 55.3 ± 18.2 vs. 45.5 ± 15.5%, *p* = 0.019), vital capacity (VC % predicted, 66.4 ± 16.2 vs. 59.0 ± 12.6, p = 0.043) and total airway resistance (Rtot 178.6 ± 104.7 vs. 248.3 ± 136.3, *p* = 0.009). Other PFT values including absolute FEV1 and VC values and blood gas analysis were similar between the groups.

**Table 2:** Pulmonary function test and laboratory findings; baseline characteristics

**Variables**	***n* overall**	**BEC < 300 n/µl *n* = 83**	**BEC ≥ 300 n/µl *n* = 24**	***p*-value**
**Pulmonary function test**				
FEV1 (% predicted)	107	55.3 ± 18.2	45.5 ± 15.5	.019* ^c^
FEV1 (l)	107	1.67 ± 0.7	1.36 ± 0.5	.050 ^c^
FEV1/FVC	107	67.0 ± 14.3	62.2 ± 13.7	.145 ^c^
VC (% predicted)	107	66.4 ± 16.2	59.0 ± 12.6	.043* ^c^
VC (l)	107	2.53 ± 1.0	2.24 ± 0.5	.063 ^d^
TLC (% predicted)	107	104.7 ± 23.8	103.3 ± 22.3	.801 ^c^
TLC (l)	107	6.63 ± 1.6	6.68 ± 1.8	.903 ^c^
RV (% predicted)	107	173.1 ± 65.9	180.6 ± 61.0	.618 ^c^
Rtot (% predicted)	107	178.6 ± 104.7	248.3 ± 136.3	.009* ^c^
DLCO (% predicted)	106	40.5 ± 21.3	46.0 ± 28.9	.312 ^c^
KCO (% predicted)	106	49.6 ± 22.3	51.7 ± 21.2	.682 ^c^
**Blood gas analysis**				
pO2 (mmHg)	106	68.3 ± 16.9	67.5 ± 7.9	.813 ^c^
pCO2 (mmHg)	106	34.7 ± 5.9	36.1 ± 7.8	.347 ^c^
pH	106	7.44 ± 0.05	7.43 ± 0.03	.247 ^c^
**Laboratory findings**				
CRP (mg/l)	89	2.5 [0.9;4.7]	3.4 [1.7;5.1]	.280 ^e^
Blood eosinophil count (n/µl)	107	154.2 ± 76.1	415.4 ± 100.6	< 0.001* ^c^
Leucocyte count (G/l)	107	7.9 ± 2.6	9.7 ± 3.0	.004* ^c^

Categorial variables are presented as n (%). Metric variables are presented as mean ± standard deviation or median [25%-percentile;75%-percentile] when not normally distributed

*Abbreviations*: *BEC* blood eosinophil count, *CRP* C-reactive protein, *DLCO* diffusing capacity for carbon monoxide, *FEV1* forced expiratory volume in one second, *FVC* forced vital capacity, *KCO* CO transfer coefficient (DLCO/alveolar volume), *l* liter, *µl* microliter, *Rtot* total airway resistance, *RV* residual volume, *TLC* total lung capacity, *VC* vital capacity

*p <.05

^c^unpaired t-test

^d^Welch test

^e^Mann-Whitney-U-test

CRP levels were generally low across all patients. The leucocyte count was higher in the high BEC group, though still within the normal range (7.9 ± 2.6 vs. 9.7 ± 3.0 G/l, *p* = 0.004).

### Strain analysis

**Fig. 3:**
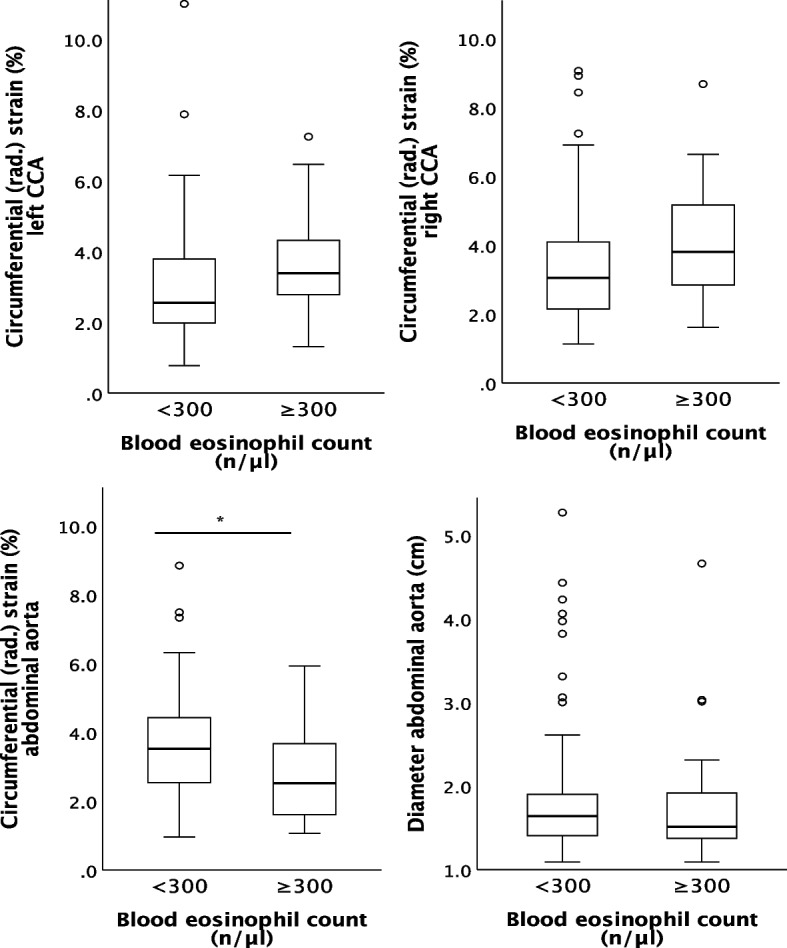
Vascular examinations of the left and right common carotid artery (CCA) and the infrarenal abdominal aorta (AA). Comparison between COPD patients with a blood eosinophil count of < and ≥ 300 n/µl via boxplots. * indicates a significant p-value of <.05. **a** Circumferential (rad.) strain of the left CCA. **b** Circumferential (rad.) strain of the right CCA. **c** Circumferential (rad.) strain of the AA. **d** Diameter of the AA

The results of vascular imaging are displayed in Table 3 and Fig. 3 a-d. Strain analysis could overall not detect any meaningful differences between the two groups, however reduced strain values of the AA for the BEC ≥ 300/µl group were detected.

**Table 3:** Vascular imaging

**Variables**	**n overall**	**BEC < 300 n/µl *n* = 83**	**BEC ≥ 300 n/µl *n* = 24**	***p*-value**
**Abdominal aorta**				
Diameter (cm)	107	1.88 ± 0.8	1.79 ± 0.8	.674 ^c^
Atherosclerotic plaque	107	29 (34.9%)	8 (33.3%)	.884 ^a^
Abdominal aortic aneurysm	107	9 (10.8%)	2 (8.3%)	1.000^b^
initial diagnosis of AAA		3 (3.6%)	0 (0.0%)	1.000 ^b^
Circumferential (rad.) strain (%)	107	3.6 ± 1.5	2.8 ± 1.4	.014* ^c^
Circumferential strain (%)	107	1.88 ± 1.4	1.46 ± 1.0	.177 ^c^
Circumferential strain rate (1/s)	107	0.15 ± 0.1	0.13 ± 0.1	.371 ^c^
Radial strain rate (1/s)	107	0.32 ± 0.1	0.32 ± 0.2	.905 ^c^
Radial displacement (mm)	107	0.16 ± 0.1	0.11 ± 0.1	.011* ^d^
Radial velocity (cm/s)	107	0.13 ± 0.1	0.10 ± 0.0	.071 ^d^
**Left common carotid artery**				
Atherosclerotic plaque	106	57 (69.5%)	17 (70.8%)	.901 ^a^
Circumferential (rad.) strain (%)	106	3.0 ± 1.6	3.6 ± 1.5	.053 ^c^
Circumferential strain (%)	106	2.65 ± 1.6	3.30 ± 2.11	.103 ^c^
Circumferential strain rate (1/s)	106	0.20 ± 0.1	0.23 ± 0.1	.207^c^
Radial strain rate (1/s)		0.25 ± 0.12	0.29 ± 0.12	.087 ^c^
Radial displacement (mm)	106	0.11 ± 0.1	0.14 ± 0.1	.081 ^c^
Radial velocity (cm/s)	106	0.09 ± 0.1	0.10 ± 0.1	.165 ^c^
**Right common carotid artery**				
Atherosclerotic plaque	106	56 (68.3%)	17 (70.8%)	.813 ^a^
Circumferential (rad.) strain (%)	106	3.5 ± 1.8	4.1 ± 1.8	.127 ^c^
Circumferential strain (%)	106	3.2 ± 1.6	3.8 ± 1.9	.153 ^c^
Circumferential strain rate (1/s)	106	0.23 ± 0.1	0.26 ± 0.1	.251 ^c^
Radial strain rate (1/s)	106	0.28 ± 0.1	0.33 ± 0.1	.110 ^c^
Radial displacement (mm)	106	0.14 ± 0.1	0.17 ± 0.1	.100 ^c^
Radial velocity (cm/s)	106	0.10 ± 0.1	0.12 ± 0.1	.174 ^c^

Categorial variables are presented as n (%). Metric variables are presented as mean ± standard deviation

*Abbreviations*: *AAA* abdominal aortic aneurysm, *BEC* blood eosinophil count, *cm* centimeter, *mm* millimeter, *µl* microliter, *s* second

^*^p <.05

^a^chi-square-test

^b^Fisher’s exact test

^c^unpaired t-test

^d^Welch test

Specifically, no difference of the circumferential (rad.) strains of the left and right CCA could be detected (*p* = 0.053 and *p* = 0.127, respectively, Fig. 3 a and b). However, the group with BEC ≥ 300/µl demonstrated reduced vascular motion of the abdominal aorta, evidenced by a lower circumferential (rad.) strain (3.6 ± 1.5 vs. 2.8 ± 1.4%, *p* = 0.014, depicted in Fig. 3 c) and reduced radial displacement (0.13 ± 0.1 vs. 0.10 ± 0.0 mm, *p* = 0.011). These were the only differences observed.

### Abdominal aortic diameter and aneurysm

The mean diameter of the AA was within the normal range and did not differ between the two groups of our cohort (1.88 ± 0.8 vs. 1.79 ± 0.8 cm, *p* = 0.674, depicted in Fig. 3 d).

Three patients were newly diagnosed with an abdominal aortic aneurysm (AAA), all from the group with a BEC < 300/µl (*p* = 1.000), with a diameter of 3.0, 4.2 and 4.9 cm. The overall prevalence of AAA was 10.8 vs. 8.3% (*p* = 1.000) (Table 3).

### Atherosclerotic plaques

The atherosclerotic plaque burden was generally high, with 34.9 vs 33.3% (*p* = 0.884) in the abdominal aorta, and approximately 70% in the left and right CCA, but no difference between high and low BEC groups could be detected (*p* = 0.901 and *p* = 0.813).

### Linear regression analysis

**Fig. 4:**
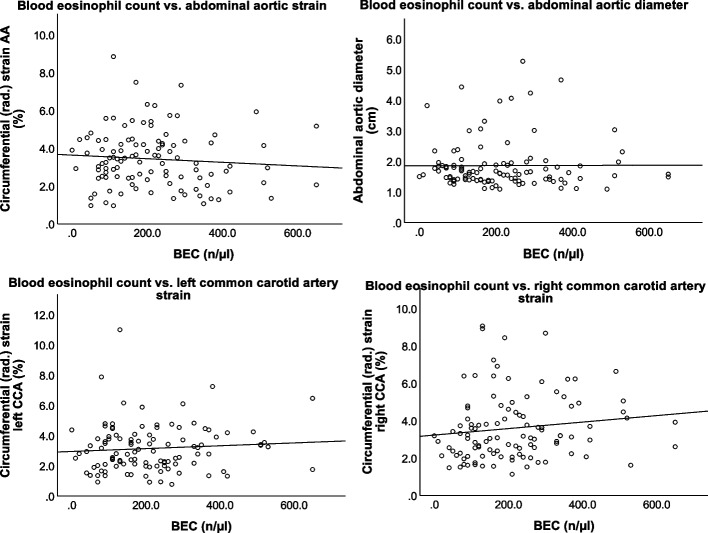
Four line graphs depicting the relationship between the blood eosinophil count (BEC) and the abdominal aortic (AA) circumferential (rad.) strain, the abdominal aortic diameter and the circumferential (rad.) strains of the left and right common carotid artery (CCA)

To further evaluate the relation between blood eosinophil count and the primary outcome variables, we assessed a linear regression analysis, adjusted for cardiovascular risk factors. It showed a significant, weak negative association between the BEC and the abdominal aortic circumferential (rad.) strain (b = −0.001 [95% CI: −0.003–0.001], *p* = 0.044). It could not detect any association between the BEC and vascular strains of the right CCA (b = −0.002 [95% CI: −0.001–0.004], *p* = 0.664), the left CCA (b = 0.001 [95% CI: −0.002–0.003], *p* = 0.576), or for the AA diameter (b = −0.000 [95% CI: −0.001–0.001], *p* = 0.672). A graphical representation Is provided in Fig. 4.

### Subgroup analyses, COPD group E

We performed a subgroup analysis of patients with COPD group E, who are at a specific risk for cardiovascular events. The results are presented at Table 4. Overall, there were no differences regarding circumferential (rad.) strain, radial strain rate, AA diameter, AAA prevalence or atherosclerotic plaque burden between the two groups of our cohort with a BEC < 300/µl or ≥ 300/µl, except a higher radial strain rate for the left CCA in patients with BEC ≥ 300/µl, but of questionable clinical relevance.

**Table 4:** Subgroup, COPD group E

**Variables**	**n overall**	**BEC < 300 n/µl *n* = 26**	**BEC ≥ 300 n/µl *n* = 12**	***p*-value**
**Abdominal aorta**				
Diameter (cm)	38	1.55 [1.38;1.84]	1.47 [1.18;1.77]	.230 ^e^
Atherosclerotic plaque	38	11 (42.3%)	4 (33.3%)	.728 ^b^
Abdominal aortic aneurysm	38	4 (15.4%)	1 (8.3%)	1.000 ^b^
Initial diagnosis of AAA	38	1 (3.8%)	0 (0.0%)	1.000 ^b^
Circumferential (rad.) strain (%)	37	3.0 [2.3;3.9]	2.5 [1.5;4.2]	.545 ^e^
Radial strain rate (1/s)	37	0.30 [0.23;0.33]	0.32 [0.18;0.43]	.653 ^e^
**Left common carotid artery**				
Atherosclerotic plaque	37	18 (72.0%)	10 (83.3%)	.687 ^b^
Circumferential (rad.) strain (%)	37	2.5 [1.9;3.6]	3.4 [2.8;4.2]	.133 ^e^
Radial strain rate (1/s)	37	0.21 [0.16;0.28]	0.31 [0.24;0.34]	.006* ^e^
**Right common carotid artery**				
Atherosclerotic plaque	37	16 (64.0%)	9 (75.0%)	.711 ^b^
Circumferential (rad.) strain (%)	37	3.1 [2.3;3.7]	3.8 [2.9;5.0]	.253 ^e^
Radial strain rate (1/s)	37	0.25 [0.21;0.32]	0.30 [0.23;0.37]	.240 ^e^

Categorial variables are presented as n (%). Metric variables are presented as mean ± standard deviation

*Abbreviations*: *AAA* abdominal aortic aneurysm, *BEC* blood eosinophil count, *cm* centimeter, *µl* microliter, *s* second

^*^p <.05

^b^Fisher’s exact test

^e^Mann-Whitney-U-test

## Discussion

Our study demonstrates no association between elevated blood eosinophil counts in COPD patients and carotid vascular strain values or aortic diameter. However, a weak negative association of BEC and abdominal aortic strain values could be detected.

COPD is frequently associated with various comorbidities, particularly cardiovascular diseases, which account for a significant proportion of the mortality observed in COPD patients [2]. Systemic inflammation, notably Type I inflammation and neutrophilic inflammation, is a recognized contributing factor to these comorbidities, extending beyond conventional risk factors such as smoking [3, 4]. Recently, eosinophilic inflammation has gained attention in the context of COPD as a treatable trait. The prevalence of eosinophilic inflammation in COPD is estimated to be between 20 and 40%, and the blood eosinophil count in COPD patients is generally higher than in healthy controls [10, 31].

The risk of cardiovascular (CV) events is especially elevated during and up to one year following acute exacerbations [32]. COPD patients experiencing exacerbations benefit more from additional inhaled corticosteroid (ICS) therapy, with a higher blood eosinophil count correlating to greater therapeutic efficacy [33, 34]. ICS therapy has been shown to reduce mortality in exacerbating COPD patients, likely driven by a reduction in cardiovascular events [34–36]. Conversely, some studies suggest an increased risk of acute exacerbations in COPD patients with higher BEC; however, the evidence for using BEC to predict future exacerbations remains insufficient, as evaluated by the GOLD committee due to conflicting study results [37].

A U.S.-american cohort study revealed an association between elevated BEC and the presence of abdominal aortic aneurysm, considered an end-stage manifestation of atherosclerotic disease [6]. Furthermore, murine studies have shown eosinophils to promote atherosclerotic plaque formation and arterial thrombosis [7]. Notably, also especially low blood eosinophils have been associated with CV events and AAA [6, 38]. However, all these studies did not specifically target COPD patients. Consequently, we focused our research on the particular subset of COPD patients with different BECs.

Our study findings, however, indicate no significant difference in vascular strains or increase in infrarenal abdominal aortic diameter, AAA or atherosclerotic plaque burden among COPD patients with BEC ≥ 300/µl. Recognizing atherosclerosis as a multifactorial and slowly progressing condition, we also employed strain analysis to detect pre-atherosclerotic changes. Despite this approach, we did not find convincing evidence of differences in vascular strains in COPD patients with elevated BEC, compared to low BEC.

A plethora of immunological studies have addressed the complex roles of T-cell subsets in atherosclerosis. While Th1-type inflammation is known to contribute to atherosclerosis, the influence of Type 2 inflammation remains unclear, with contradictory study results, which have been summarized by Saigusa et al. [39]. Some research even suggests a protective role for Type 2 interleukins, such as IL-5 and IL-13 [39]. More definitive evidence exists for the eosinophil cationic protein (ECP), which has been repeatedly identified as a cardiovascular risk factor that promotes atherogenesis [40, 41]. The underlying pathophysiological mechanisms are complex and not fully understood, necessitating further research, which was beyond the scope of our current study.

### Limitations

Based on the exploratory nature of this study, it is important to note that only hypotheses can be generated, and no causal interactions can be definitively postulated. The study's monocentric design, conducted at a university hospital, introduces a potential selection bias, as it is likely that a sicker patient population was included. All participants, as well as those in both the proband and control groups, have life-threatening diseases at varying stages of progression. Given the multifactorial nature of atherosclerosis, a larger patient cohort may be necessary to detect significant differences between the groups. Further on, strain analysis is a common but not guideline-implemented method for measuring vascular motion, and there are multiple methods for measuring (pre-)atherosclerotic changes that we have not used, e.g. intima-media-thickness. Also, blood pressure as a driver of vascular motion is currently unjustly neglected in the standard practice of vascular strain measurements, we have addressed this issue by excluding a difference in mean blood pressure. Nevertheless, this harbours a source of error that requires further studies, but this was not the subject of our study. Additionally, a limitation is the reliance on a single measurement of eosinophil count, which can vary due to multiple influencing factors. To mitigate this variability, only eosinophil counts obtained during periods without oral steroid therapy and without exacerbations were used.

## Data Availability

Data is provided within the manuscript or supplementary information files, all other data is available from the authors upon reasonable request.

## Abbreviations

AA: Abdominal aorta
AAA: Abdominal aortic aneurysm
ATS: American Thoracic Society
BEC: Blood eosinophil count
BGA: Blood gas analysis
CAT: COPD Assessment Test
CCA: Common carotid artery
CRP: C-reactive protein
COPD: Chronic obstructive pulmonary disease
CV: Cardiovascular
ECG: Electrocardiography
EGPA: Eosinophilic granulomatosis with polyangiitis
ERS: European Respiratory Society
FEV1: Forced expiratory volume in one second
GLI: Global Lung Initiative
GOLD: Global Initiative for Chronic Obstructive Lung Disease
ICS: Inhaled corticosteroid
IQR: Interquartile range
LABA: Long-acting beta-2-agonist
LAMA: Long-acting muscarinic antagonist
LTOT: Long-term oxygen therapy
NVL: “Nationale Versorgungsleitlinie” (German National Clinical Practice Guideline)
PFT(s): Pulmonary function test(s)
QoL: Quality of life
Rtot: Total airway resistance
SD: Standard deviation
VC: Vital capacity
WHO: World Health Organization
